# The leaky pipeline of diverse race and ethnicity representation in academic science and technology training in the United States, 2003–2019

**DOI:** 10.1371/journal.pone.0284945

**Published:** 2023-04-26

**Authors:** Ashish Sarraju, Summer Ngo, Fatima Rodriguez

**Affiliations:** 1 Department of Cardiovascular Medicine, Heart, Vascular, and Thoracic Institute, Cleveland Clinic, Cleveland, Ohio, United States of America; 2 Division of Cardiovascular Medicine and Cardiovascular Institute, Stanford University School of Medicine, Stanford, California, United States of America; Emory University School of Medicine, UNITED STATES

## Abstract

**Introduction:**

Diverse race and ethnicity representation remains lacking in science and technology (S&T) careers in the United States (US). Due to systematic barriers across S&T training stages, there may be sequential loss of diverse representation leading to low representation, often conceptualized as a leaky pipeline. We aimed to quantify the contemporary leaky pipeline of S&T training in the US.

**Methods:**

We analyzed US S&T degree data, stratified by sex and then by race or ethnicity, obtained from survey data the National Science Foundation and the National Center for Science and Engineering Statistics. We assessed changes in race and ethnicity representation in 2019 at two major S&T transition points: bachelor to doctorate degrees (2003–2019) and doctorate degrees to postdoctoral positions (2010–2019). We quantified representation changes at each point as the ratio of representation in the later stage to earlier stage (representation ratio [RR]). We assessed secular trends in the representation ratio through univariate linear regression.

**Results:**

For 2019, the survey data included for bachelor degrees, 12,714,921 men and 10.612,879 women; for doctorate degrees 14,259 men and 12,860 women; and for postdoctoral data, 11,361 men and 8.672 women. In 2019, we observed that Black, Asian, and Hispanic women had comparable loss of representation among women in the bachelor to doctorate transition (RR 0.86, 95% confidence interval [CI] 0.81–0.92; RR 0.85, 95% CI 0.81–0.89; and RR 0.82, 95% CI 0.77–0.87, respectively), while among men, Black and Asian men had the greatest loss of representation (Black men RR 0.72, 95% CI 0.66–0.78; Asian men RR 0.73, 95% CI 0.70–0.77)]. We observed that Black men (RR 0.60, 95% CI 0.51–0.69) and Black women (RR 0.56, 95% CI 0.49–0.63) experienced the greatest loss of representation among men and women, respectively, in the doctorate to postdoctoral transition. Black women had a statistically significant decrease in their representation ratio in the doctorate to postdoctoral transition from 2010 to 2019 (p-trend = 0.02).

**Conclusion:**

We quantified diverse race and ethnicity representation in contemporary US S&T training and found that Black men and women experienced the most consistent loss in representation across the S&T training pipeline. Findings should spur efforts to mitigate the structural racism and systemic barriers underpinning such disparities.

## Introduction

Representation of diverse race and ethnicity groups in the science and technology (S&T) workforce remains lacking in the United States (US) despite research and governmental recognition of the problem [1–5]. Black, Hispanic, American Indian and Native Alaskan, and Native Hawaiian and Pacific Islander groups together comprise only 10% of the federal S&T workforce despite substantially higher representation in the general population according to the 2020 US Census: 12.4% (Black), 32.7% (Hispanic), 1.1% (American Indian and Native Alaskan [AI/AN]), and 0.1% (Native Hawaiian and Pacific Islander) [4]. The “leaky pipeline” framework conceptualizes low representation based on “leaks” due to systemic barriers across the education and training continuum [1].

Prior work using data from 2000 to 2013 assessed the journey of Black, Hispanic, and AI/AN populations across the training pathway from high school to research faculty positions in the biomedical sciences in the US, finding losses of representation mainly during undergraduate studies, with otherwise minimal losses of attrition in the receipt of a doctorate degree or receipt of a postdoctoral position [3]. Updated trends in representation since 2013, aggregated trends across S&T fields, trends by sex, as well as representation of Asian populations who represent one of the fastest growing groups in the US, remain incompletely understood. To address these gaps, we sought to quantify the contemporary leaky pipeline of US S&T training by race and ethnicity groups across three major training stages: undergraduate (bachelor) degrees, doctorate degrees, and postdoctoral positions.

## Methods

We obtained cross-sectional 2003–2019 S&T (which included all reported science and engineering fields) degree data from the National Science Foundation and the National Center for Science and Engineering Statistics which was comprised of findings from the National Survey of College Graduates, the Survey of Doctorate Recipients, and the Survey of Graduate Students and Postdoctorates in Science and Engineering (2010–2019). Race and ethnicity groups were included based on available data: Non-Hispanic White (NHW), Non-Hispanic Black (Black), Non-Hispanic Asian (Asian), Hispanic or Latino (Hispanic), and Non-Hispanic AI/AN. In NCSES survey data, those classified as Hispanic or Latino may be of any race, while those categorized as a race exclude those of Hispanic ethnicity. Temporary visa holders were excluded from the analysis because their race or ethnicity data are not reported. We calculated race or ethnicity group representation (%) separately for men and women and evaluated two transition points: bachelor to doctorate degree receipt, and doctorate degree receipt to postdoctoral positions. We quantified differences in representation at each transition as the ratio of representation in the subsequent stage to that of the previous stage in the same year. A representation ratio less than 1 indicated loss of representation across that transition point. We calculated 95% confidence intervals (CIs) for the representation ratios in the standard manner as for risk ratios. AI/AN data were excluded due to very low sample sizes (for example, N<50 for doctoral data) with consequent limited interpretability and reliability. We assessed trends over time in representation ratios using univariate linear regression. A p-trend threshold of 0.05 denoted statistical significance. R, version 4.0.0, was used for statistical analyses.

## Results

**Fig 1:**
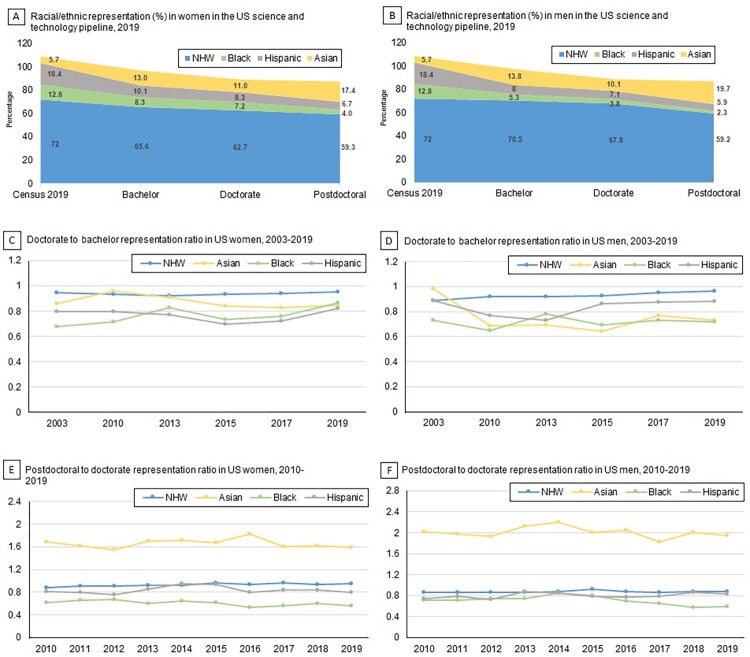
Race and ethnicity representation in the US science and technology pipeline, 2003–2019. Panel A–Race and ethnicity representation among US women in bachelor, doctorate, postdoctoral positions in 2019 versus US Census estimates; Panel B–Race and ethnicity representation among US men in bachelor, doctorate, postdoctoral positions in 2019, versus US Census estimates; Panel C–Ratios of doctorate to bachelor representation by race and ethnicity for US women, 2003–2019, all p-trend > 0.05; Panel D–Ratios of doctorate to bachelor representation by race and ethnicity for US men, 2003–2019, NHW men p-trend = 0.004 (ratio change per year 0.004 [95% CI 0.002,0.007]), all other p-trend > 0.05; Panel E—Ratios of postdoctoral to doctorate representation by race and ethnicity for US women, 2010–2019, NHW women p-trend = 0.007 (ratio change per year 0.006 [95% CI 0.002, 0.01]), all p-trend > 0.05; Black women p-trend = 0.02 (ratio change per year -0.01 [95% CI -0.02, -0.002]), all other p-trend > 0.05; Panel F–Ratios of postdoctoral to doctorate representation by race and ethnicity for US men, 2010–2019, all p-trend > 0.05. For panels A-B, US Census estimates refer to population-level estimates from the 2019 American Community Survey, all sexes combined. For panels C-F, ratios are obtained by dividing the representation of the race and ethnicity group in the subsequent stage by representation in the previous stage. A ratio less than 1 suggests loss of representation. Native Hawaiian and Pacific Islanders not visualized to due to incomplete data availability. AI/AN groups are not visualized due to representation <0.5%. Abbreviations: CI, confidence interval; NHW, Non-Hispanic White.

In 2019, the survey data included the following total populations: for bachelor degrees, 12,714,921 men and 10.612,879 women; for doctorate degrees 14,259 men and 12,860 women; and for postdoctoral data, 11,361 men and 8.672 women. In 2019, NHW men (bachelor 70.5%, doctorate 67.8%, postdoctoral 59.2%) and women (bachelor 65.6%, doctorate 62.7%, postdoctoral 59.3%) had the highest representation, followed by Asian, Hispanic, and Black groups (Fig 1A and 1B).

### Bachelor to doctorate transition

In 2019, Black, Asian, and Hispanic women had comparable losses in representation (ratios 0.86, 95% confidence interval [CI] 0.81–0.92; 0.85, 95% CI 0.81–0.89; and 0.82, 95% CI 0.77–0.87, respectively), while NHW women had the lowest change (ratio 0.96, 95% CI 0.94–0.97, Fig 1C). Among men, Black (ratio 0.72, 95% CI 0.66–0.78) and Asian men (ratio 0.73, 95% CI 0.70–0.77) had the greatest loss of representation, while NHW men had the lowest change (ratio 0.96, 95% CI 0.95–0.97, Fig 1E). From 2003 to 2019, NHW men had a small increase in the representation ratio (p = 0.004), with no statistically significant trends for other groups among men or women (all p > 0.05, Fig 1C and 1D).

### Doctorate to postdoctoral transition

In 2019, among women, Black women demonstrated the greatest loss of representation (ratio 0.56, 95% CI 0.49–0.63, Fig 1E). Among men, Black men demonstrated the greatest loss of representation (ratio 0.60, 95% CI 0.51–0.69, Fig 1F). From 2010 to 2019, NHW women had a small increase in the representation ratio (p = 0.007), while Black women experienced a small decrease (p = 0.02), with no statistically significant trends for other groups among men or women (all p > 0.05, Fig 1E and 1F).

## Discussion

Black men and women experienced the most consistent losses in representation across the aggregated contemporary US S&T training pipeline from bachelor degrees to postdoctoral positions. The leaky aggregated S&T pipeline has largely remained unchanged across diverse race and ethnicity groups in men and women from 2003 to 2019, with Black women experiencing a possibly small decrease in representation in the transition from doctoral degree receipt to postdoctoral positions.

As race and ethnicity generally represent sociocultural constructs, observed disparities likely reflect structural racism and barriers. Prior work suggested that in the biomedical sciences, from 2000 to 2013, loss of representation in underrepresented minority (URM) groups defined as Black, Hispanic, and AI/AN groups was minimal in the receipt of doctoral degrees and the receipt of a postdoctoral position within the biomedical sciences [3]. Our data from a more recent period at an aggregated S&T level suggest losses of representation across these transitions in certain groups when disaggregated by sex. Additionally, our analysis utilizes a simple, intuitive variable–the representation ratio–to quantify “leaks” in the training pipeline (that is, loss of representation at each stage of transition) and demonstrate that this variable can potentially be tracked over time to quantify progress or lack of progress in improving the leaky pipeline. Efforts are needed to further explore and validate these findings and address the biases underpinning the leaky S&T pipeline.

This analysis does not capture other transitions including high school to undergraduate or postdoctoral to faculty. The pipeline concept does not incorporate all S&T careers; for instance, postdoctoral training may not be relevant to non-tenure track academic or industry positions, or entrepreneurial careers. Heterogeneity by disaggregated groups or disaggregated fields of study was not captured in these data. Higher Asian representation may reflect decreasing representation in other groups and should be interpreted with caution. Those on temporary visas were not captured in this analysis. These data aim to explore longitudinal trends using serial cross-sectional survey data and should be interpreted within these limitations.

In conclusion, Black men and women experienced the greatest representation losses across the aggregated US S&T training pipeline from bachelor degrees to postdoctoral positions with no significant evidence of improvement over a contemporary time period.
